# The effect of hydroxychloroquine on cholesterol synthesis depends on the profile of cholesterol metabolism. A controlled clinical study

**DOI:** 10.1016/j.athplu.2024.02.002

**Published:** 2024-03-01

**Authors:** Piia Simonen, Lotta Ulander, Kari K. Eklund, Mikko Niemi, Janne T. Backman, Helena Gylling, Juha Sinisalo, OXI pilot trial

**Affiliations:** a Heart and Lung Center, Cardiology, University of Helsinki and Helsinki University Hospital, Helsinki, Finland; b Department of Rheumatology, Helsinki University Hospital, Helsinki University, ORTON Orthopaedic Hospital of the Orton Foundation, Helsinki, Finland; c Department of Clinical Pharmacology and Individualized Drug Therapy Research Program, Faculty of Medicine, University of Helsinki, Finland; d Department of Clinical Pharmacology HUS Diagnostic Center, Helsinki University Hospital, Helsinki, Finland

**Keywords:** Cholesterol metabolism, Lanosterol, Desmosterol, Hydroxychloroquine, Myocardial infarction

## Abstract

**Background and aims:**

Hydroxychloroquine (HCQ) has a variable effect on cholesterol synthesis. To clarify this, we assessed the effect of HCQ on the cholesterol-synthesis pathway in individuals with low and high cholesterol absorption efficiency.

**Method:**

A total of 53 acute myocardial infarction patients with a constant statin dose randomized to receive HCQ or placebo for six months in a double-blind manner, were classified further into low (n = 26) and high (n = 27) cholesterol absorbers based on the median baseline serum cholestanol level. Serum lipids and biomarkers of cholesterol synthesis (squalene, lanosterol, zymostenol, desmosterol, and lathosterol) and absorption efficiency (sitosterol and cholestanol), were measured at baseline and one-, six-, and 12-month follow-up visits.

**Results:**

In low cholesterol absorbers, serum cholesterol concentration and cholesterol synthesis and absorption biomarkers did not differ between the HCQ and placebo groups. At one month, high cholesterol absorbers with HCQ had lower serum cholesterol concentration and serum lanosterol to cholesterol ratio in comparison to the placebo group (HCQ 3.18 ± 0.62 vs. placebo 3.71 ± 0.65, p = 0.042, and HCQ 10.4 ± 2.55 vs. placebo 13.1 ± 2.36, p = 0.008, respectively). At 12 months, serum desmosterol to cholesterol ratio was lower in HCQ users (HCQ 47.1 ± 7.08 vs. placebo 59.0 ± 13.1, p = 0.011).

**Conclusions:**

HCQ affects the cholesterol-synthesis pathway in high cholesterol absorbers. It reduces serum lanosterol and desmosterol ratios and consequently serum cholesterol concentration possibly by inhibiting the activity of lanosterol synthase as described earlier in *vitro* studies.

**Trial registration:**

ClinicalTrials.gov Identifier: NCT02648464.

## Highlights

• Myocardial infarction patients received HCQ or placebo for six months.
• Patients were subdivided into low and high cholesterol absorbers.
• HCQ affected the cholesterol synthesis in high cholesterol absorbers.
• At one month, serum cholesterol and lanosterol were lower in high absorbers.
• Serum desmosterol was lower in high absorbers at twelve months.

## Graphical abstract

**Figure:**
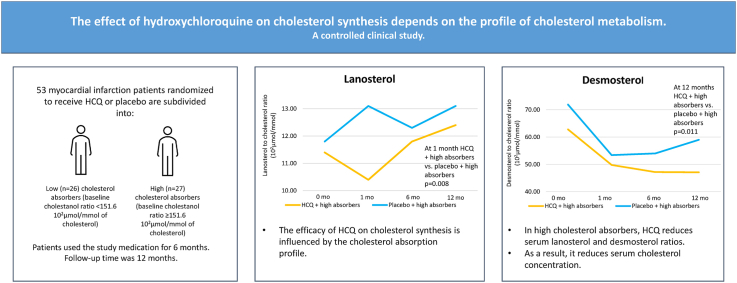

## 1 Introduction

Hydroxychloroquine (HCQ), a derivative of chloroquine (CQ), is a widely used antirheumatic drug with anti-inflammatory, antiviral, immunomodulatory, and lipid-lowering characteristics, the latter especially in subjects with rheumatoid arthritis and systemic lupus erythematosus [[1], [2], [3]]. The combination of its anti-inflammatory and cholesterol-lowering capacity encouraged us to evaluate in a randomized, placebo-controlled, double-blind OXI trial whether HCQ combined with statins prevents atherosclerosis and reduces the risk of cardiovascular events in patients with acute myocardial infarction (MI) [4,5]. HCQ reduced plasma interleukin-6 concentrations compared to placebo [5] but did not affect serum-, low-density lipoprotein cholesterol (LDL-C) or high-density lipoprotein cholesterol (HDL-C) concentrations [6]. However, HCQ interfered with the cholesterol precursors lanosterol, zymostenol, and desmosterol ratios to cholesterol at different time points [6].

In *vitro* studies, CQ inhibited the activity of lanosterol synthase (EC 5.4.99.7) and reduced the cellular concentrations of squalene, lanosterol, and desmosterol [7]. Zymostenol was not analyzed. Plausibly these results apply also to HCQ because of the almost analogical pharmacokinetic properties of CQ and HCQ [8]. Thus, the inhibition of lanosterol synthase could also explain the effects of HCQ on serum cholesterol precursors in the OXI trial [6]. However, the different profiles of whole-body cholesterol metabolism (low absorption - high synthesis vs. high absorption - low synthesis of cholesterol) affect not only cholesterol homeostasis but possibly also the development of atherosclerosis [[9], [10], [11], [12]]. Thus, the type of cholesterol metabolism might also have an impact on cholesterol-lowering by HCQ.

In this OXI substudy, we investigated whether the profile of cholesterol metabolism brings additive information on the effect of HCQ on the cholesterol-synthesis pathway, especially on serum lanosterol-, zymostenol-, and desmosterol to cholesterol ratios observed earlier [6]. The population was classified into low and high cholesterol absorbers based on the median serum cholestanol or phytosterol ratio of the study population at baseline, a generally used method in population-based studies (e.g., [9], [13], [14]). Serum cholestanol and phytosterol ratios are validated biomarkers of cholesterol absorption efficiency [[15], [16], [17], [18], [19]].

## 2 Materials and methods

### 2.1 Patients

The original design of the OXI trial was published previously [4,5]. Briefly, 125 MI patients were randomized in a median of 43 h after hospitalization to receive either HCQ 300 mg/day (weight <60 kg: 300 mg for five days/week) or placebo for six months. Atorvastatin 40–80 mg/day was started after MI and was continued for 12 months. Morning EDTA plasma samples were taken at baseline, one month, six months, and 12 months.

To the present OXI substudy, 53 patients originally randomized to placebo (n = 25) and HCQ (n = 28) groups and whose plasma concentrations of atorvastatin, HCQ, and their metabolites were available at every time point, were selected from the previous study [6]. They were divided into low (n = 26) and high (n = 27) cholesterol absorber subgroups based on baseline cholestanol ratio (baseline cholestanol ratio <151.6 and ≥ 151.6 10^2^ μmol/mmol of cholesterol) (Table 1).

**Table 1:** Demographics, serum cholesterol and noncholesterol sterol levels, plasma concentrations of atorvastatin, HCQ^a^, and their active metabolites in individuals with low and high cholesterol absorption efficiency in the HCQ and placebo groups.

Variables	Low cholesterol absorption, n = 26			High cholesterol absorption, n = 27		
	HCQ group n = 17	Placebo group n = 9	p-value	HCQ group n = 11	Placebo group n = 16	p-value
***Baseline***						
Gender, females/males, n	6/11	1/8	0.201	1/10	1/15	0.792
Age, years	57.3 ± 8.02	57.2 ± 9.27	0.970	58.7 ± 8.73	54.4 ± 10.3	0.270
Body mass index, kg/m^2^	30.3 ± 5.68	30.4 ± 5.08	0.980	26.3 ± 3.84	27.3 ± 5.87	0.642
Cholesterol, mmol/L	5.33 ± 1.18	4.68 ± 1.13	0.191	4.31 ± 0.96	4.91 ± 0.89	0.108
Lanosterol^b^	11.3 ± 2.72	10.4 ± 3.63	0.476	11.4 ± 2.85	11.8 ± 2.29	0.639
Zymostenol^b^	11.3 ± 5.84	9.93 ± 2.58	0.496	8.36 ± 3.45	9.00 ± 3.40	0.637
Desmosterol^b^	75.1 ± 18.7	72.9 ± 16.2	0.772	62.8 ± 29.0	71.9 ± 19.9	0.338
Lathosterol^b^	68.4 ± 57.5	53.4 ± 21.7	0.461	36.4 ± 20.3	43.7 ± 26.1	0.442
Sitosterol^b^	97.9 ± 31.1	98.7 ± 24.0	0.953	161 ± 62.1	143 ± 52.7	0.412
Cholestanol^b^	135 ± 13.1	134 ± 14.6	0.826	181 ± 18.7	182 ± 25.2	0.949
Atorvastatin and 2-hydroxy atorvastatin, ng/ml	45.3 ± 39.8 n = 17	40.4 ± 26.8 n = 9	0.741	47.8 ± 44.6 n = 11	59.8 ± 39.2 n = 16	0.469
**1 month**						
Cholesterol, mmol/L	3.20 ± 0.57	3.12 ± 0.39	0.742	3.18 ± 0.62	3.71 ± 0.65	0.042
Lanosterol^b^	12.5 ± 3.88	10.9 ± 2.21	0.253	10.4 ± 2.55	13.1 ± 2.36	0.008
Zymostenol^b^	7.98 ± 1.92	7.51 ± 1.52	0.525	6.26 ± 1.07	6.60 ± 1.46	0.516
Desmosterol^b^	55.3 ± 11.9	56.6 ± 8.62	0.791	49.8 ± 11.1	53.4 ± 11.4	0.427
Lathosterol^b^	33.0 ± 13.2	39.4 ± 13.3	0.255	27.2 ± 13.2	26.5 ± 8.70	0.860
Sitosterol^b^	208 ± 78.0	205 ± 53.4	0.906	316 ± 202	299 ± 125	0.784
Cholestanol^b^	186 ± 33.9	180 ± 47.2	0.694	224 ± 32.5	227 ± 39.0	0.821
Atorvastatin and 2-hydroxy atorvastatin, ng/ml	42.2 ± 26.4 n = 17	36.9 ± 23.3	0.369	39.2 ± 24.0 n = 11	41.8 ± 23.4	0.783
HCQ and metabolites, ng/ml^c^	741 (126–1923) n = 17			682 (142–3825) n = 11		
**6 months**						
Cholesterol, mmol/L	3.46 ± 0.69	3.33 ± 0.52	0.623	3.30 ± 0.52	3.55 ± 0.77	0.356
Lanosterol^b^	12.4 ± 2.92	11.4 ± 1.95	0.368	11.8 ± 2.83	12.3 ± 2.28	0.624
Zymostenol^b^	7.79 ± 2.60	7.94 ± 2.46	0.889	6.73 ± 1.64	7.47 ± 1.93	0.309
Desmosterol^b^	54.0 ± 18.2	55.7 ± 14.0	0.805	47.2 ± 10.5	54.0 ± 10.9	0.114
Lathosterol^b^	32.7 ± 13.6	37.2 ± 11.6	0.412	27.4 ± 11.5	29.4 ± 12.8	0.668
Sitosterol^b^	251 ± 109	256 ± 77.8	0.907	336 ± 132	336 ± 166	0.995
Cholestanol^b^	196 ± 42.3	200 ± 26.3	0.836	234 ± 38.2	241 ± 46.8	0.687
Atorvastatin and 2-hydroxy atorvastatin, ng/ml	34.4 ± 32.9 n = 17	37.4 ± 33.0	0.829	32.1 ± 15.8 n = 11	44.1 ± 30.3	0.242
HCQ and metabolites, ng/ml^c^	199 (18–2045) n = 17			476 (37–2621)^4^ n = 10		
**12 months**						
Cholesterol, mmol/L	3.53 ± 0.59	3.39 ± 0.56	0.568	3.53 ± 0.64	3.86 ± 0.87	0.289
Lanosterol^b^	11.1 ± 2.51	11.2 ± 2.56	0.901	12.4 ± 3.37	13.1 ± 3.32	0.604
Zymostenol^b^	8.32 ± 2.30	8.93 ± 2.46	0.536	7.02 ± 1.16	8.26 ± 2.72	0.168
Desmosterol^b^	53.9 ± 13.0	56.0 ± 7.38	0.657	47.1 ± 7.08	59.0 ± 13.1	0.011
Lathosterol^b^	36.3 ± 11.8	42.9 ± 16.5	0.251	29.8 ± 11.2	34.7 ± 11.2	0.278
Sitosterol^b^	251 ± 127	255 ± 46.6	0.930	316 ± 154	294 ± 174	0.738
Cholestanol^b^	190 ± 38.5	191 ± 31.1	0.906	228 ± 36.8	231 ± 54.9	0.876
Atorvastatin and 2-hydroxy atorvastatin, ng/ml	39.8 ± 21.6	26.5 ± 16.1	0.117	38.2 ± 29.1	53.3 ± 57.8	0.431
HCQ and metabolites, ng/ml^c^	13 (6–37)(n = 6)			12 (7–32)(n = 5)		

Mean ± SD, median (range).

^4^p-value = 0.021 between the groups of low and high cholesterol absorption. Low cholesterol absorption efficiency: serum cholestanol ratio <151.6 10^2^ μmol/mmol, high cholesterol absorption efficiency: serum cholestanol ratio ≥151.6 10^2^ μmol/mmol of cholesterol.

a HCQ = hydroxychloroquine.

b 10^2^ μmol/mmol of cholesterol.

c Sum of desethyl HCQ, desethyl chloroquine, and didesethyl chloroquine concentrations.

The OXI trial was approved by the Ethics Committee of Helsinki University Hospital (Approval number: 148/13/03/01/2015) and conducted in accordance with the Declaration of Helsinki. The trial was registered on ClinicalTrials.gov Identifier: NCT02648464. All patients gave written informed consent. The study was organized, coordinated, and executed by researchers at the Heart and Lung Center at Helsinki University Hospital, who were also responsible for data management and statistical analysis.

## 3 Methods

Serum cholesterol, cholesterol precursors squalene, lanosterol, zymostenol, desmosterol, and lathosterol, and sitosterol, a plant sterol, and cholestanol, a metabolite of cholesterol, were analyzed using gas–liquid chromatography (GLC) with a 50-m capillary column (Ultra 2, Agilent Technologies, Wilmington, DE) and flame ionization detection with 5α-cholestane as internal standard [20]. The samples from different time points per subject were analyzed in the same GLC run. Serum concentrations of the noncholesterol sterols were adjusted to that of cholesterol and expressed as ratios to cholesterol. Serum cholesterol precursor ratios are biomarkers of cholesterol synthesis, and sitosterol and cholestanol ratios are biomarkers of cholesterol absorption efficiency validated e.g., to absolute measurements of cholesterol absorption and synthesis and to variants of genes of cholesterol absorption and synthesis [[15], [16], [17], [18], [19],^21^].

The concentrations of plasma atorvastatin and its active metabolite 2-hydroxy atorvastatin [22], and plasma HCQ and its active metabolites desethyl HCQ, desethyl chloroquine, and didesethyl chloroquine were determined as described earlier [6]. Plasma concentrations of atorvastatin and 2-hydroxy atorvastatin were combined as well as the concentrations of HCQ and its active metabolites. The therapeutic plasma level of HCQ and its metabolites is ≥ 500 ng/ml [23].

### 3.1 Statistics

Statistical analyses were performed by using SPSS for Windows 25.0 (SPSS, Chicago, IL). Sample size calculation was based on significance levels (α = 0.05 and β = 0.20). Essential information was obtained from previous studies [2,3]. Using these estimates, the size of the required population was appropriate. Normality and homogeneity of variance assumptions were checked, and variables not normally distributed were transformed logarithmically. Continuous variables were tested by using the Student's t-test. The groups were compared with independent samples *t*-test. Non-continuous variables were analyzed with Fisher's exact chi-square test. The results are expressed as mean ± SD and median (range).

## 4 Results

The study population involved nine females and 44 males (Table 1). Even though the number of women was smaller than men in both low and high cholesterol absorbers, the gender distribution did not differ between the HCQ and placebo groups within the low and high cholesterol absorbers (Table 1). Age and body mass index (BMI) did not differ between the HCQ and placebo groups within the low and high cholesterol absorbers at baseline. BMI remained unchanged throughout the intervention.

In low cholesterol absorbers (n = 26), the mean baseline serum cholestanol ratio was 135 ± 13.3 (SD), and in high cholesterol absorbers (n = 27) 181 ± 22.4 10^2^ μmol/mmol of cholesterol (p < 0.001 between the groups). Serum sitosterol ratio was also higher in high (150 ± 56.3) vs. low (98.2 ± 28.3 10^2^ μmol/mmol of cholesterol) cholesterol absorbers (p < 0.001 between the groups). Serum lathosterol ratio was lower in high (40.8 ± 23.8) vs. low (63.2 ± 48.1 10^2^ μmol/mmol of cholesterol) cholesterol absorbers (p < 0.001 between the groups). Serum cholestanol and lathosterol ratios correlated inversely with each other (e.g., high cholesterol absorption, HCQ group, n = 11, r = −0.872, p < 0.001, and low cholesterol absorption, HCQ group, n = 17, r = −0.505, p < 0.05) indicating steady state in the study population, validity of the biomarkers, and supporting proper classification between low and high cholesterol absorbers [18].

In low cholesterol absorbers, serum cholesterol concentration and all biomarker ratios were similar in the HCQ and placebo groups throughout the study (Table 1).

In high cholesterol absorbers at one month, serum cholesterol concentration and serum lanosterol ratios were significantly lower from the baseline values in the HCQ (cholesterol, p < 0.002, lanosterol, p < 0.05) and placebo groups (cholesterol, p < 0.001, lanosterol p < 0.02), and both values were significantly lower in the HCQ vs. placebo group (Table 1). In low cholesterol absorbers, these variables did not significantly differ between the HCQ and placebo groups. At 12 months, serum desmosterol ratio was lower in the HCQ vs. placebo group. Serum squalene or zymostenol ratios were similar between the groups (data not shown).

Serum sitosterol and cholestanol ratios were similar in the HCQ and placebo groups in both low and high cholesterol absorbers (Table 1). They were higher in the high vs. low cholesterol absorbers as expected.

The mean atorvastatin dose was 66.4 ± 14.7 (SD) mg/day in the HCQ group at baseline and one and six months, and 67.9 ± 18.0 mg/day at 12 months (ns between the time points), and in the placebo group 73.6 ± 14.7 mg/day during each time point (ns between the HCQ and placebo groups).

The sum of plasma concentrations of atorvastatin and its active metabolite was similar between the HCQ and placebo groups in the low and high cholesterol absorbers at every time point (Table 1). The sum of the plasma concentrations of HCQ and its active metabolites varied; the concentrations were highest at one month and the median levels exceeded the therapeutic level. At six months, the median concentrations were higher in the high vs. low cholesterol absorbers (p = 0.021), but at 12 months only traces were present in both groups. Plasma HCQ + metabolites did not correlate with serum cholesterol absorption biomarkers.

## 5 Discussion

The new observations were, first, that the profile of cholesterol metabolism, i.e., low absorption - high synthesis vs. high absorption - low synthesis of cholesterol influenced the effect of HCQ on serum cholesterol and its precursor sterol levels. In high cholesterol absorbers, serum cholesterol concentration and lanosterol and desmosterol ratios were lower in the HCQ vs. placebo group.

Second, the results suggest that HCQ inhibits the activity of lanosterol synthase also in humans. Low serum lanosterol and desmosterol ratios in high cholesterol absorbers with HCQ resembled the earlier *in vitro* findings with CQ [7] and the results in our previous human study [6]. Unlike the previous studies, HCQ did not affect serum squalene and zymostenol ratios [6,7]. In low cholesterol absorbers, HCQ did not interfere with the cholesterol-synthesis pathway. A possible explanation is that low cholesterol absorption-activated increase in cholesterol synthesis overpowered the inhibitory capacity of HCQ on lanosterol synthase. Lathosterol was not affected by CQ or HCQ [6,7]. Regarding the lack of effect on zymostenol and lathosterol, one possibility is that the localizations of the enzymes affected by HCQ in the cholesterol synthesis pathway are strictly regulated.

Third, in high cholesterol absorbers, HCQ lowered serum cholesterol by interfering with the cholesterol-synthesis pathway. It cannot be ruled out that in high cholesterol absorbers, HCQ might also delay the absorption of cholesterol and cholesterol precursors, or simply its higher concentration explains the more powerful effect.

In high cholesterol absorbers, HCQ reduced serum cholesterol concentration and lanosterol and desmosterol ratios at one and 12 months compared with the placebo group. Remarkably, these results were similar to those obtained in the OXI trial but now even more convincing for the lanosterol ratio [6], since lanosterol differed significantly between the HCQ and placebo groups.

Low desmosterol ratio at 12 months in the HCQ group's high cholesterol absorbers corresponds to the earlier findings [6]. The constant and similar serum concentrations of atorvastatin and its metabolite between the groups ruled out their role in decreasing the desmosterol ratio. Despite the low serum concentrations of HCQ and metabolites at 12 months, their long half-lives could enable the inhibition of lanosterol synthase and lower serum desmosterol ratio in the HCQ group in high cholesterol absorbers even after six months of the cessation of HCQ use.

### 5.1 Limitations of the study

Even though the number of participants originally was large regarding the complexity of the long-term clinical intervention, the number of individuals was occasionally limited. According to power calculations, the number of individuals was sufficient, and the results regarding the lower serum ratios of lanosterol and desmosterol in the HCQ vs. placebo group were in agreement with our previous study of the larger OXI trial [6].

Regarding the classification of the study population into low and high cholesterol absorbers, we used the baseline median value of serum cholestanol ratio of the whole study population. Using either serum phytosterol or serum cholestanol ratio is a method generally used in population-based studies to classify the rate of cholesterol absorption efficiency (e.g., [9], [13], [14], [18]).

There are some caveats concerning the serum biomarkers of cholesterol metabolism. They are relative indicators of cholesterol metabolism, and even though their validity is generally accepted, it is not self-evident in different populations, and they should be validated in ‘new’ populations. During consumption of phytosterol-enriched diets, serum phytosterols do not adequately reflect cholesterol absorption efficiency.

In the present study, the significant inverse correlations between the biomarkers of cholesterol absorption and cholesterol synthesis confirmed the intact homeostasis of cholesterol metabolism, the steady state of the study population, and the validity of the serum biomarkers. In addition, the similar atorvastatin doses throughout the study in the HCQ and placebo groups and the similar plasma concentrations of atorvastatin and its active metabolite between the two groups and in the low and high cholesterol absorbers at every time point suggest that atorvastatin did not confound the results.

Discrepancies between the serum biomarkers and absolute measurements of cholesterol metabolism are rare. In one study serum phytosterol ratio did not correlate with the absorption efficiency of cholesterol, even though serum cholestanol ratio did correlate with it [24]. The mean cholesterol absorption efficiency was also exceptionally low, 24 ± 14 (SD)%, compared with a mean value of 56 ± 12 (SD)% in another study using the same absolute methods in the assessment of the absorption efficiency of cholesterol [25]. The reasons for these discrepant results possibly resulted from the complicated methods assessing the metabolism of cholesterol [18].

## 6 Conclusions

The profile of cholesterol metabolism influenced the efficacy of HCQ on cholesterol metabolism. HCQ affected the cholesterol-synthesis pathway only in high cholesterol absorbers. Thus, the profile of cholesterol metabolism may have a more powerful influence not only on the development and progression of cholesterol-related diseases but also on the efficacy of cholesterol-lowering drugs.

### Funding

The funding has been raised from the 10.13039/100010133Aarne Koskelo Foundation, 10.13039/501100003125Finnish Cultural Foundation, 10.13039/501100005633Finnish Foundation for Cardiovascular Research, Finnish Academy of Sciences, and 10.13039/501100008484Paavo Nurmi Foundation. The trial received special government funds and a non-restricted grant from the 10.13039/100004325Finnish Cardiac Society/AstraZeneca. Orion Pharma provided the trial medication.

### Data availability

Additional data supporting the findings of this study can be requested from the corresponding author.

## Author information

The following investigators and institutions participated in the trial: Heart and Lung Center, Helsinki University Hospital, University of Helsinki, Helsinki, Finland: LU, PS, KKE, HG, and JS. Department of Rheumatology, Helsinki University Hospital, Helsinki University, ORTON Orthopaedic Hospital of the Orton Foundation, Helsinki, Finland: KKE, Department of Clinical Pharmacology and Individualized Drug Therapy Research Program, Faculty of Medicine, University of Helsinki: MNI and JTB.

## Declaration of competing interest

The authors declare the following financial interests/personal relationships which may be considered as potential competing interests: Juha Sinisalo reports personal fees from Abbott, Amgen, Bayer, and a grant from AstraZeneca. Janne T. Backman and Mikko Niemi report consultant fees from Orion Pharma.
